# FOXO1 regulates osteogenic differentiation of periodontal ligament stem cells through the METTL3 signaling pathway

**DOI:** 10.1186/s13018-023-04120-w

**Published:** 2023-08-29

**Authors:** Qi Wang, Wei Shi, Shaozhan Lin, Hanxue Wang

**Affiliations:** 1 Foshan Dengte Dental Clinic, Fenjiang Middle Road, Chancheng District, Foshan, 528000 China; 2 MeiQi Dental Clinic, Wuhan Mengya Dentistry, Wuhan, 430000 China

**Keywords:** FOXO1, METTL3, Periodontitis, PDLSCs, Osteogenic differentiation

## Abstract

**Background:**

Periodontitis is a chronic inflammation that occurs in periodontal tissue and has a high incidence rate. Periodontal ligament stem cells (PDLSCs) are ideal candidates for periodontal tissue and bone regeneration in patients with periodontitis. The purpose of this work was to analyze the molecular mechanisms that affect the osteogenic differentiation of PDLSCs.

**Methods:**

In this work, qRT‒PCR was used to detect the mRNA expression level of FOXO1 in clinical tissues and PDLSCs. Alkaline phosphatase (ALP) staining and Alizarin red S (ARS) staining were used to detect the degree of osteogenic differentiation of PDLSCs. qRT‒PCR and western blotting were used to measure the levels of the early osteogenic markers COL1A1 and RUNX2. The JASPAR online database was used to predict FOXO1-regulated genes.

**Results:**

FOXO1 was generally expressed at low levels in clinical samples from patients with periodontitis. We provided evidence that overexpression of FOXO1 promoted osteogenic differentiation in PDLSCs. In addition, both in vitro and rescue experiments showed that FOXO1 regulated METTL3. FOXO1 affected osteogenic differentiation mainly by regulating METTL3 modification of the PI3K/AKT pathway.

**Conclusions:**

FOXO1 activated the PI3K/AKT signaling pathway by transcriptionally activating METTL3. This effect promoted the osteogenic differentiation of PDLSCs.

## Introduction

Periodontitis is a chronic inflammation that occurs in periodontal tissue [1]. This disease is caused by microorganisms (such as *Streptococcus mutans*, *Porphyromonas gingivalis*, and *Staphylococcus*) [2]. The accumulation of these microbial plaques destroys the connective cells and tissues around the teeth and can also cause inflammatory reactions in the oral cavity [3]. In recent years, the prevalence of periodontitis worldwide has approached 50%. Moreover, the average initial age of periodontitis has decreased from 35 years old to 30 years old [4]. The fourth national oral health epidemiological survey in China showed that less than 10% of adults in China have periodontal health, with moderate and severe periodontitis patients accounting for 62.4% [5]. Patients with advanced periodontitis often experience multiple symptoms, such as tooth loosening, gum bleeding, and periodontal abscess. In severe cases, tooth loss can occur [6]. Therefore, exploration of the pathology of periodontitis is particularly important.

Periodontal ligament stem cells (PDLSCs) are stem cells derived from periodontal ligaments. These cells have the ability to self-renew and show multidirectional differentiation potential [7]. PDLSCs have the ability to differentiate into periodontal nerve cells, blood vessels, periodontal ligaments, and bone tissue [8, 9]. PDLSCs are considered the best candidates for periodontal bone regeneration. Controlling the differentiation of PDLSCs is also considered an effective method for the clinical treatment of patients with advanced periodontitis [10]. Therefore, for the clinical treatment of periodontitis patients, identification of drugs and drug targets that promote the osteogenic differentiation of PDLSCs is urgently needed [11]. FOXO1 transcription factors belong to the Fox family. FOXO1 is involved in cartilage differentiation, diabetes, oesophageal cancer and other diseases [12–14]. Previous studies have shown that FOXO1 can promote the osteogenic differentiation of PDLSCs. However, the complete mechanism of the regulatory process is unclear [15, 16].

N^6^-methyladenosine (m^6^A) modification is one of the most abundant transcriptomic modifications of RNA [17]. This modification has two biological effects when applied to mRNA. On the one hand, due to structural changes, the modified mRNA binds more closely to the target protein. On the other hand, specific proteins are recruited to bind to target RNA. These processes are all completed by m^6^A-modifying enzymes (METTL3, METTL14, ALKBH5, etc.) [18]. The m^6^A methylation modification is closely related to the differentiation process of stem cells. The methylation modification of m^6^A can affect the maintenance, differentiation, migration, and other cellular functions of stem cells [19–21]. In addition, it has been proposed that m^6^A methylation modification can promote the osteogenic differentiation of PDLSCs in patients with periodontitis [22].

The PI3K/AKT signaling pathway can regulate the self-renewal and pluripotent differentiation of stem cells. Among them, pluripotent differentiation includes osteogenic regeneration of stem cells. The PI3K/AKT pathway has been extensively explored in many diseases, such as cancer, osteoporosis, and fractures [23–25]. Many studies have shown that the PI3K/AKT signaling pathway promotes osteogenic differentiation in PDLSCs [26]. Moreover, other studies have shown that the m^6^A-modifying enzyme METTL3 mediates PI3K/AKT signaling pathways to promote stem cell function changes [27, 28]. However, these studies have focused more on pyroptosis or cell damage. In periodontitis, research on the complete molecular mechanism and influence mode of the m^6^A-modifying enzyme-mediated PI3K/AKT signaling pathway regulating the osteogenic differentiation of PDLSCs is still incomplete [29].

Here, we investigated the mechanism by which FOXO1 regulates the osteogenic differentiation of PDLSCs. Overexpression of FOXO1 has been confirmed in clinical samples and osteogenic differentiated PDLSCs. Both in vitro cell experiments and rescue experiments showed that FOXO1 promoted the osteogenic differentiation of PDLCs by transcriptionally activating METTL3. This process was related to the PI3K/AKT signaling pathway.

## Materials and methods

### Clinical sample collection

The clinical periodontal ligament (PDL) tissue of the periodontal group was extracted from patients with severe periodontitis who came to our hospital for treatment. The clinical tissue of the normal group was extracted from the PDL of healthy individuals. Approximately 300 mg of PDL tissue was collected from each subject. All participants were informed of the experimental process of this study before sample extraction. They all signed the informed consent form. This study fully followed *the Helsinki Declaration*. In addition, all processes of this study were supervised and approved by the Orthodontics Committee of our hospital.

### PDLSC culture and osteogenic differentiation induction

The clinical organization of the PDL was selected from the root PDL of patients with periodontitis in our hospital (approximately 1 mm^3^). After the PDL tissue of the patient was extracted, 2 mg/mL collagenase and 4 mg/mL dispase (Merck, Darmstadt, Germany) in a ratio of 1:1 were added and digested at 37 ℃ for 40 min. Single-cell suspension culture was carried out in α-MEM growth medium containing 10% fetal bovine serum (Thermo Fisher Scientific, CA, USA) and 1% penicillin/streptomycin (Thermo Fisher Scientific). The conditions of the incubator were 37 ℃ and moist air with 5% CO_2_ added. PDLSCs were subcultured using 0.25% trypsin. The PDLSCs used in this study were all 2nd to 5th generation.

Osteogenic differentiation induction: The OriCell® Human Related Stem Cell Osteogenic Differentiation Induction Kit was used to induce osteogenic differentiation of PDLSCs. The experimental operation was carried out in full accordance with the manufacturer's instructions.

### Identification of hPDLSCs by flow cytometry

The hPDLSCs were incubated in 6-well plates. Then, 1 × 10^6^ cells were collected after digestion. After two washes with PBS, the supernatant was discarded, and the cells were resuspended and divided into tubes, with 500 μl per tube. The cells were centrifuged at 112 g for 5 min. After that, the supernatant was discarded. After resuspension, anti-human monoclonal phycoerythrin-labeled CD73 and CD105 antibodies and fluorescein isothiocyanate-labeled CD44 and CD90 antibodies were added to each tube. After 30 min of incubation at 4 °C in the dark, the surface markers of these four antibodies were detected by flow cytometry.

### Cell transfection

Overexpression vectors for FOXO1 (FOXO1), siFOXO1, siMETTL3, and their controls (vector and siNC) were synthesized by GenePharma (Shanghai, China). Lipofectamine™ 3000 (Thermo Fisher Scientific) was used to transfect the vectors into PDLSCs. Before transfection, the cells should be in a logarithmic growth phase.

### qRT‒PCR

TRIzol reagent (Invitrogen, Shanghai, China) was used to extract total RNA from PDLSC tissues and cells. The purity and concentration of the RNA were analyzed using a NanoDrop ND-1000 spectrophotometer (Thermo Fisher Scientific, Inc.) at an optical density of 260/280 nm. SuperScript IV reverse transcriptase was used to perform reverse transcription of total RNA. A CellsDirect™ one-step kit was used to perform qRT‒PCR. The reaction conditions were as follows (40 cycles): 95℃ for 30 s, 95℃ for 15 s, and 60℃ for 30 s. Finally, an ABI StepOne PLUS qRT‒PCR instrument (Thermo Fisher Scientific) was used for qRT‒PCR detection. GAPDH-normalized mRNA expression values were used. The relative RNA expression was calculated using the 2^−ΔΔCt^ method. The primers involved in this experiment were as follows:

- FOXO1-F: 5'-TGATAACTGGAGTACATTTCGCC-3';
- FOXO1-R: 5'-CGGTCATAATGGGTGAGAGTCT-3';
- COL1A1-F: 5'-GAGGGCCAAGACGAAGACATC-3';
- COL1A1-R: 5'-CAGATCACGTCATCGCACAAC-3';
- RUNX2-F: 5'-TCAACGATCTGAGATTTGTGGG-3';
- RUNX2-R: 5'-GGGGAGGATTTGTGAAGACGG-3';
- METTL3-F: 5'-CATTGCCCACTGATGCTGTG-3';
- METTL3-R: 5'-AGGCTTTCTACCCCATCTTGA-3';
- GAPDH-F: 5'-ATTGTTGCCATCAATGACCC-3';
- GAPDH-R: 5'-AGTAGAGGCAGGGATGATGT-3'.

### Western blot

RIPA lysis and extraction buffers (Thermo Scientific) were used to extract total proteins from PDLSCs. SDS‒PAGE was used to isolate 20 μg of total protein. Total protein was transferred to PVDF membranes (Beyotime, Shanghai, China). Five percent bovine serum albumin (BSA, Sigma Aldrich) was used as a block at room temperature for 30 min. The membrane and the primary antibodies were then incubated overnight at 4 ℃ (GAPDH was used as an internal reference). After the secondary antibody was added, the mixture was incubated at room temperature for another hour. Enzyme-labeled proteins were developed with a BeyoECL Star Kit (Beyotime), washed with TBST 3 times, and then photographed. The antibodies in this study were purchased from Abcam (MA, USA). The protein band density was measured using ECL plus reagents (Millipore, Billerica, MA, USA) and ImageJ software (Version 1.48u, Bethesda, USA).

### Alkaline phosphatase (ALP) staining and quantification

PDLSCs were cultured in medium containing glycerophosphate sodium (5 mmol/L), vitamin C (50 g/mL), dexamethasone (100 mmol/L), and FBS (10%) for osteogenic differentiation induction for 14 days. Then, the TRAP/ALP staining kit (Whatman, Maidstone, UK) was used for ALP staining. According to the manufacturer's instructions, PDLSCs were cultured on a 12-well plate and fixed with 4% paraformaldehyde for 30 min. TRAP/ALP staining buffer was then added and incubated in a dark room at room temperature for 2 h. The degree of ALP staining density was measured by spectrophotometry at 405 nm.

### Alizarin red S (ARS) staining and activity

ARS staining was performed on the 14th day after osteogenic differentiation induction of PDLSCs. PDLSCs were cultured on a 12-well plate and fixed with 4% paraformaldehyde for 30 min. Then, the cells were stained with 1% ARS solution (pH = 8.4) (Merck) for 5 min. After three washes with PBS, 10 mM sodium phosphate containing 10% cetylpyridine chloride was used to extract the dye. Finally, the degree of staining was measured by spectrophotometry at 562 nm.

### Chromatin immunoprecipitation (ChIP) and ChIP‒qPCR

The cells were cleaved after being fixed with formaldehyde. A chromatin extraction kit (AB 117152, Abcam) was used to prepare chromatin. A Diagenode Bioruptor Pico Ultrasonic crusher (Belgium) was used to shear chromatin to an average size of 250–300 bp (approximately 25 cycles). The ChIP Kit Magnetic—One Step (ab156907, Abcam) was used for ChIP analysis. Chromatin was mixed with antibodies or IgG in ChIP buffer according to the manufacturer's instructions. The mixture was washed and then eluted with DNA release buffer and protease K. The samples were crosslinked in reverse (15 min at 65 °C and then 10 min at 95 °C). After DNA was purified, the enrichment of NEK7 was analyzed by qRT‒PCR.

### Bioinformatics analysis

The JASPAR online database (https://jaspar.genereg.net/) was used to predict the DNA-binding motif of the transcription factor FOXO1.

### Statistical analysis

The significance of the difference was determined through one-way ANOVA or Student’s t test. The Spearman correlation coefficient was used to calculate the correlation between the two groups. GraphPad Prism (version 8.0) and SPSS (version 17.0) software were used for statistical analysis. All data are expressed as the mean ± standard deviation. *p* < 0.05 indicated a statistically significant difference.

## Results

### Low expression of FOXO1 in clinical tissues of periodontitis

**Fig. 1:**
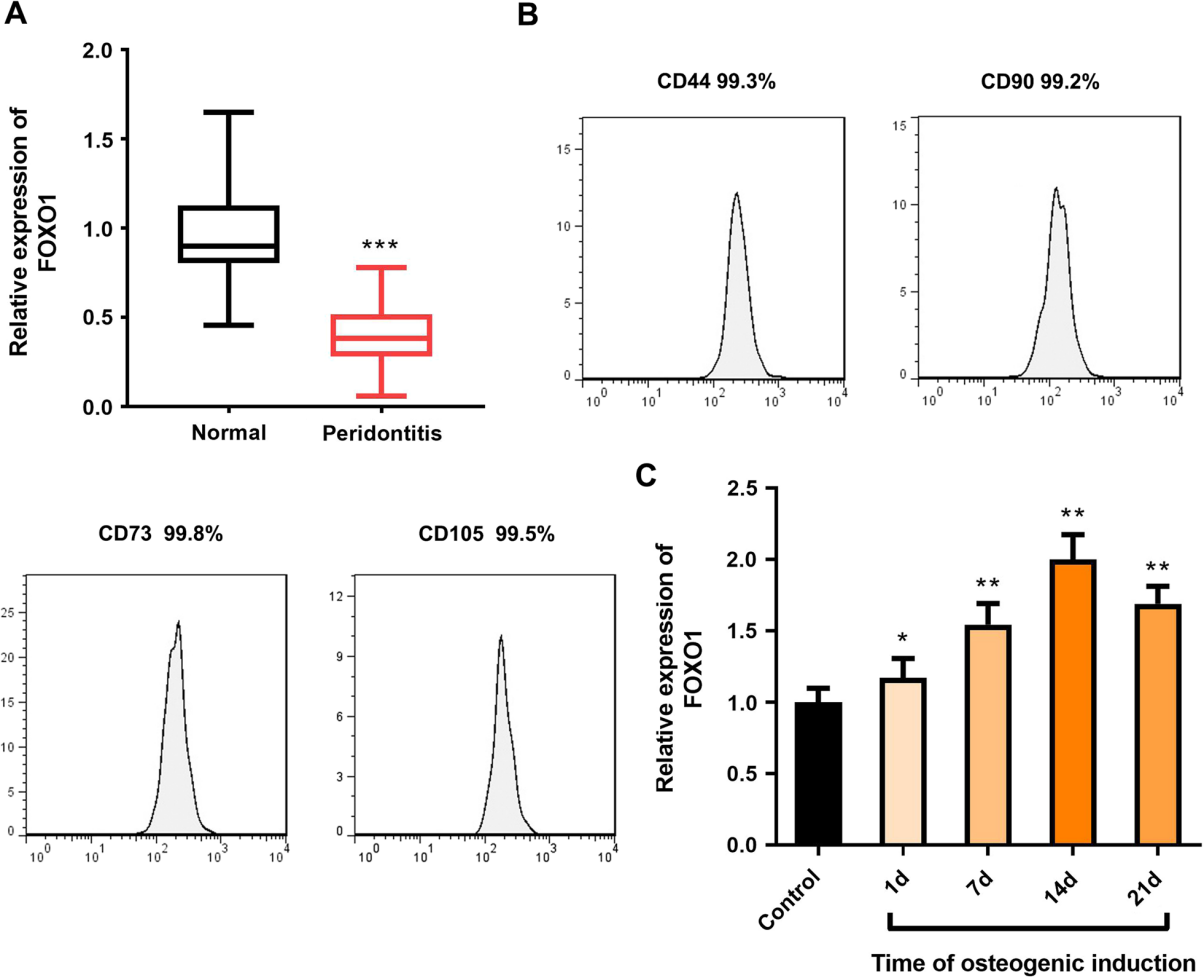
Clinical expression of FOXO1 and expression in PDLSCs during osteogenic differentiation. **A** The expression level of FOXO1 in the periodontal ligament of patients in the periodontitis group and normal group was detected by RT‒qPCR. **B** Identification of PDLSCs by flow cytometry. **C** Expression level of FOXO1 expression during osteogenic differentiation of PDLSCs. was detected by RT‒qPCR. *p < 0.05, **p < 0.01, ***p < 0.001

To investigate the abnormal expression and role of the transcription factor FOXO1 in periodontitis, we tested the expression of FOXO1 in clinical samples from patients with periodontitis. As shown in Fig. 1A, the expression of FOXO1 in the clinical samples of the periodontitis group was significantly lower than that in the normal group. Then, we obtained PDLSCs from PDL tissues. The results of flow cytometry showed that PDLSC markers (CD73, CD90, CD105, CD44) were all positive, which indicated that we successfully obtained PDLSCs (Fig. 1B). Then, through the osteogenic differentiation of PDLSCs, we found that FOXO1 expression was increased on the 21st day of osteogenic differentiation of PDLSCs, and it peaked on the 14th day (Fig. 1C). These results suggested that the abnormal expression of FOXO1 was associated with periodontitis. The expression of FOXO1 changed with the osteogenic differentiation of PDLSCs in patients with periodontitis. Therefore, 14-day osteogenic differentiation induction conditions were applied for subsequent expression.

### Overexpression of FOXO1 promotes osteogenic differentiation of PDLSCs

**Fig. 2:**
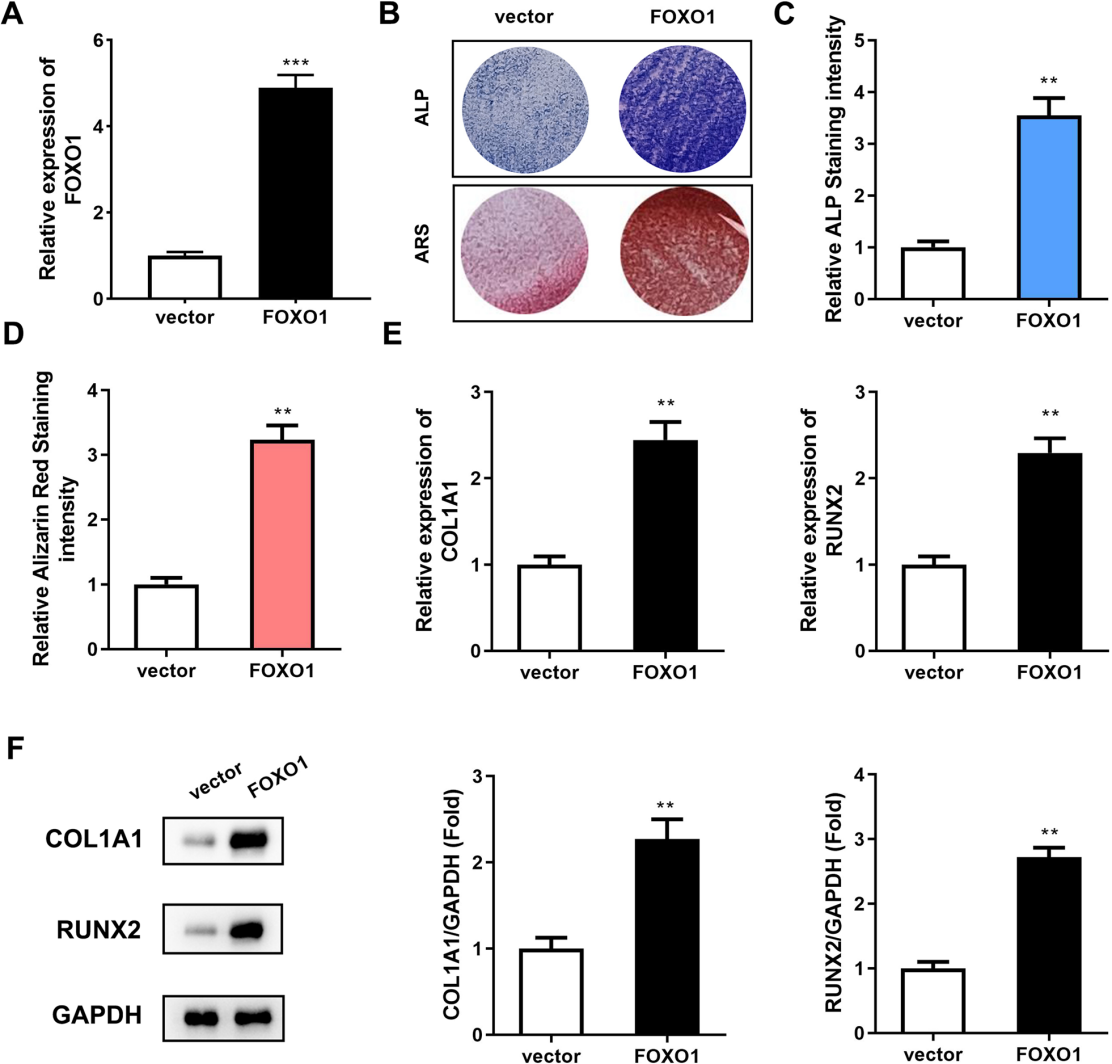
Overexpression of FOXO1 promoted osteogenic differentiation of PDLSCs. **A** The transfection efficiency of FOXO1 overexpression was measured by RT‒qPCR. The cells underwent 14 days of osteogenic differentiation induction. After FOXO1 overexpression, the osteogenic differentiation ability of the PDLSCs was detected by ALP staining (**B**, **C**) and ARS staining (**D**). **E** The mRNA expression levels of COL1A1 and RUNX2 were measured by qRT‒PCR. **F** Western blotting was used to detect the protein expression levels of COL1A1 and RUNX2. **p < 0.01, ***p < 0.001

To investigate the role of FOXO1 in the osteogenic differentiation of PDLSCs, we overexpressed FOXO1 in PDLSCs (Fig. 2A). The results of ALP staining experiments showed that the marker enzymes of mature osteoblasts were more enriched in the PDLSCs overexpressing FOXO1 than in the controls (Fig. 2B). The qualitative results of ALP staining also confirmed this phenomenon (Fig. 2C). The results of ARS staining also indicated that ARS staining of FOXO1-overexpressing PDLSCs was more intense (Fig. 2D). Thus, FOXO1 overexpression in PDLSCs increased extracellular calcium deposition nodules. In short, these two staining experiments confirmed that PDLSCs overexpressing FOXO1 have better osteogenic abilities. Therefore, we examined the mRNA and protein expression levels of the early osteogenic markers COL1A1 and RUNX2 in PDLSCs. The qRT‒PCR and western blot results showed that when FOXO1 was overexpressed, the mRNA and protein expression levels of COL1A1 and RUNX2 were higher (Fig. 2E, F). These results indicated that overexpression of FOXO1 promotes osteogenic differentiation of PDLSCs.

### FOXO1 transcription activates METTL3

**Fig. 3:**
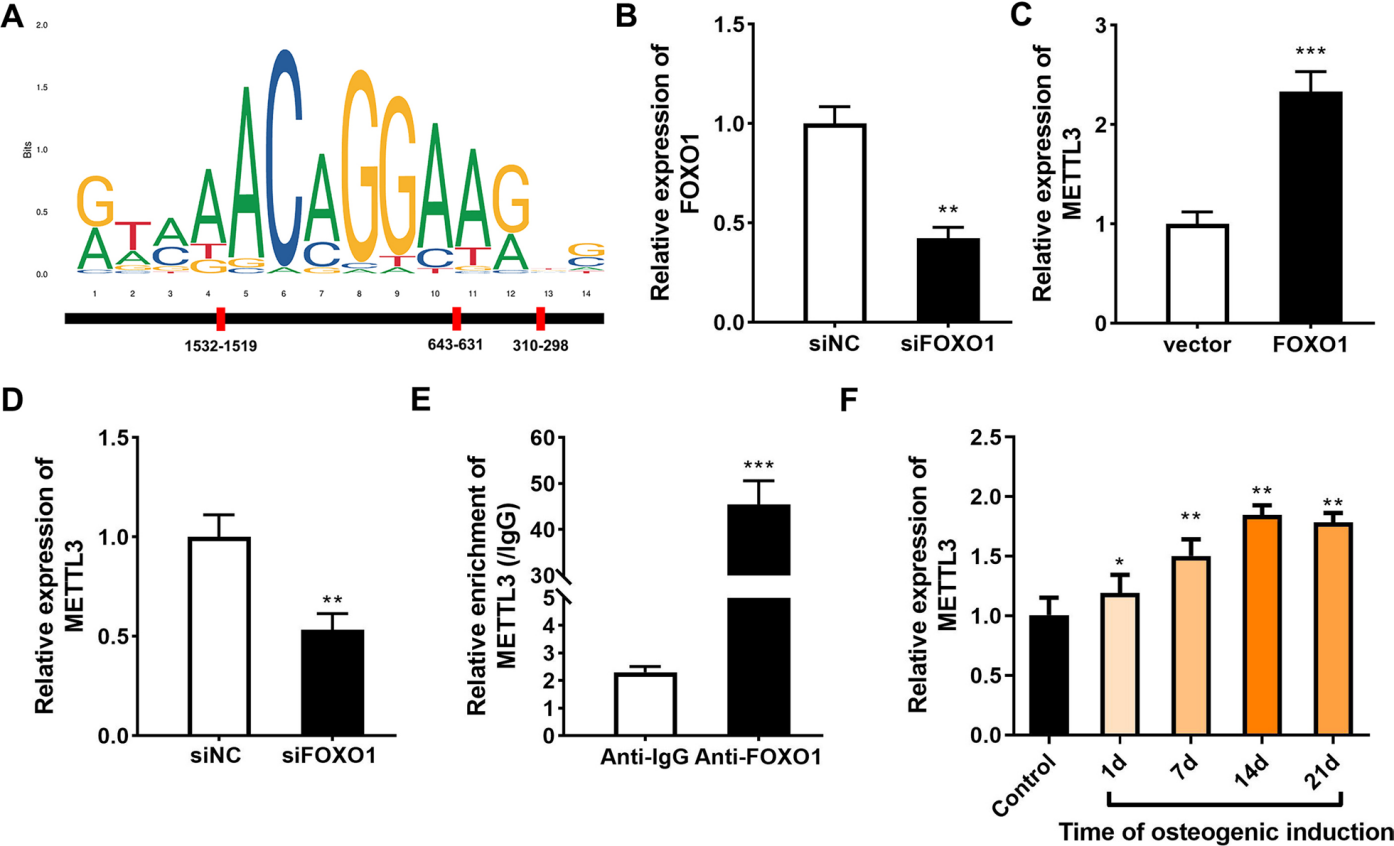
Binding effect of FOXO1 and METTL3. **A** The online software JASPAR predicted the binding protein of FOXO1. **B** The transfection efficiency of si-FOXO1 was measured by qRT‒PCR. The METTL3 levels after FOXO1 overexpression (**C**) and knockdown (**D**) were measured by qRT‒PCR. **E** ChIP analysis of METTL3 enrichment after anti-FOXO1 transfection. **F** Expression of METTL3 during osteogenic differentiation of PDLSCs was detected by RT‒qPCR. *p < 0.05, **p < 0.01, ***p < 0.001

To further clarify the mechanism by which FOXO1 promotes the osteogenic differentiation of PDLSCs, we analyzed the binding protein of the transcription factor FOXO1 through the online database JASPAR. As shown in Fig. 3A, we found 3 binding sites between the transcription factor FOXO1 and METTL3. After siFOXO1 knockdown, FOXO1 levels were significantly decreased (Fig. 3B). FOXO1 overexpression significantly increased METTL3 levels (Fig. 3c), while FOXO1 knockdown significantly decreased METTL3 levels (Fig. 3D). Furthermore, the ChIP‒qPCR experimental results showed that FOXO1 could bind to the METTL3 promoter (Fig. 3E). Similarly, METTL3 expression was increased on the 21st day of osteogenic differentiation of PDLSCs and peaked on the 14th day (Fig. 3F).

### Overexpression of METTL3 promotes osteogenic differentiation of PDLSCs

**Fig. 4:**
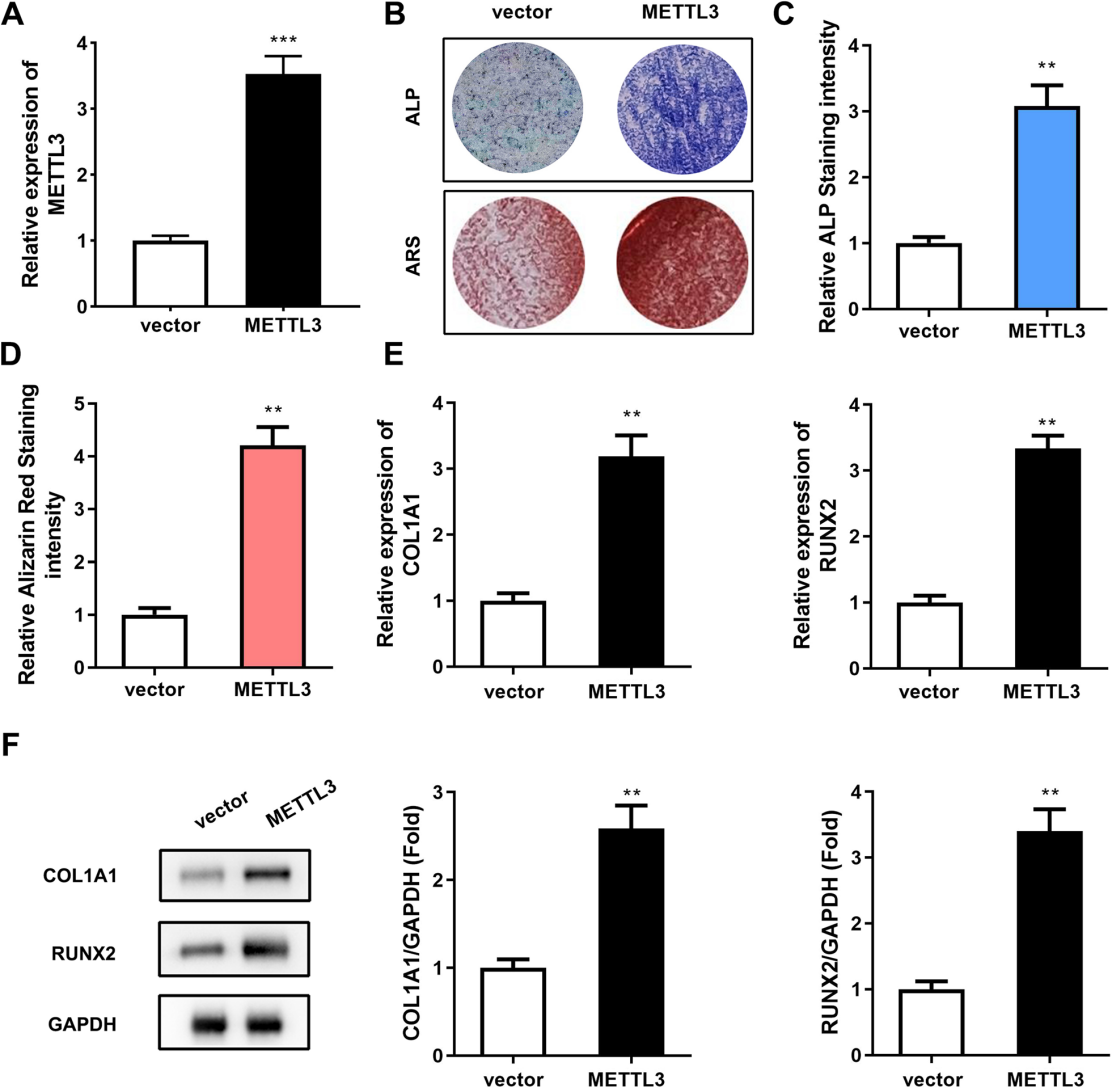
Overexpression of METTL3 promoted osteogenic differentiation of PDLSCs. **A** The transfection efficiency of METTL3 overexpression was measured by RT‒qPCR. The cells underwent 14 days of osteogenic differentiation induction. After METTL3 overexpression, the osteogenic differentiation ability of the PDLSCs was detected by ALP staining (**B**, **C**) and ARS staining (**D**). **E** The mRNA expression levels of COL1A1 and RUNX2 were measured by qRT‒PCR. **F** Western blotting was used to detect the protein expression levels of COL1A1 and RUNX2. **p < 0.01, ***p < 0.001

To investigate the role of METTL3 in the osteogenic differentiation of PDLSCs, we overexpressed METTL3 in PDLSCs (Fig. 4A). The results of ALP staining experiments showed that the marker enzymes of mature osteoblasts were more enriched in the PDLSCs overexpressing METTL3 than in the controls (Fig. 4B). The qualitative results of ALP staining also confirmed this phenomenon (Fig. 4C). The results of ARS staining also indicated that ARS staining of the METTL3-overexpressing PDLSCs was more intense (Fig. 4D). Thus, METTL3 overexpression in PDLSCs increased extracellular calcium deposition nodules. In short, these two staining experiments confirmed that PDLSCs overexpressing METTL3 have better osteogenic abilities. Therefore, we examined the mRNA and protein expression levels of the early osteogenic markers COL1A1 and RUNX2 in PDLSCs. The qRT‒PCR and western blot results showed that when METTL3 was overexpressed, the mRNA and protein expression levels of COL1A1 and RUNX2 were higher (Figs. 2F, 4E). These results indicated that overexpression of FOXO1 promotes osteogenic differentiation of PDLSCs.

### FOXO1 regulates METTL3 to promote osteogenic differentiation of PDLSCs

**Fig. 5:**
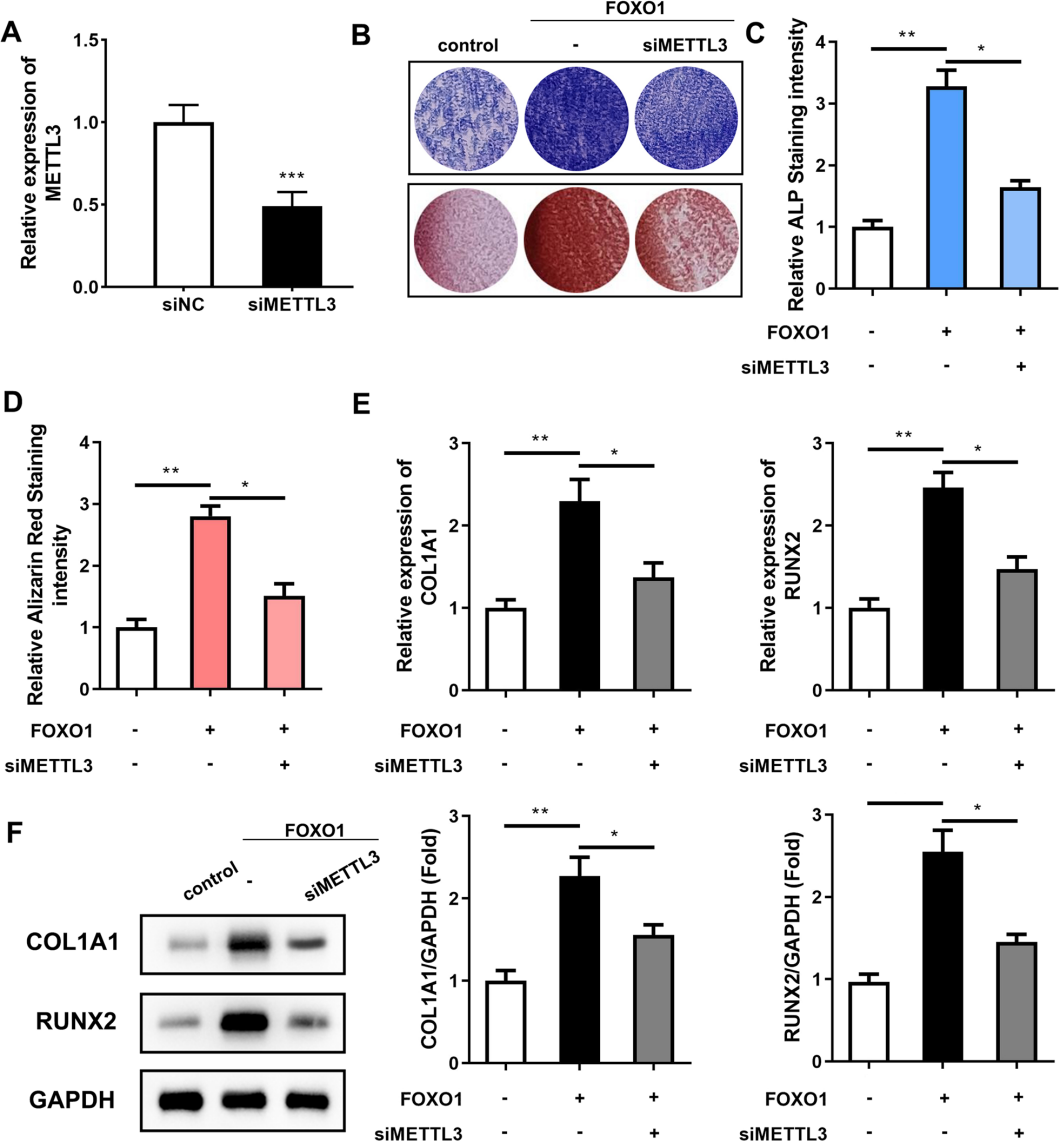
FOXO1-mediated promotion of the osteogenic differentiation of PDLSCs was modified by METTL3. **A** The transfection efficiency of siMETTL3 was measured by qRT‒PCR. The cells underwent 14 days of osteogenic differentiation induction. After FOXO1 overexpression and METTL3 knockdown, the osteogenic differentiation of the PDLSCs was detected by ALP staining (**B**, **C**) and ARS staining (**D**). **E** The mRNA expression levels of COL1A1 and RUNX2 were measured by qRT‒PCR. **F** Western blotting was used to detect the protein expression levels of COL1A1 and RUNX2. **p < 0.01, ***p < 0.001

To confirm whether FOXO1 affected the osteogenic differentiation of PDLSCs by regulating METTL3, we successfully knocked down METTL3 in PDLSCs (Fig. 5A). Our previous research results showed that the intensity of ALP staining and ARS staining was increased when FOXO1 was overexpressed. However, when METTL3 was knocked down simultaneously, the two staining intensities decreased (Fig. 5B–D). Similarly, the qRT‒PCR and western blot results showed that overexpression of FOXO1 with METTL3 knockdown inhibited the mRNA and protein expression of the early osteogenic markers COL1A1 and RUNX2 (Figs. 4F, 5E). These results indicated that the FOXO1/METTL3 axis promoted the osteogenic differentiation of PDLSCs.

### FOXO1 regulates METTL3 to promote the PI3K/AKT signaling pathway

**Fig. 6:**
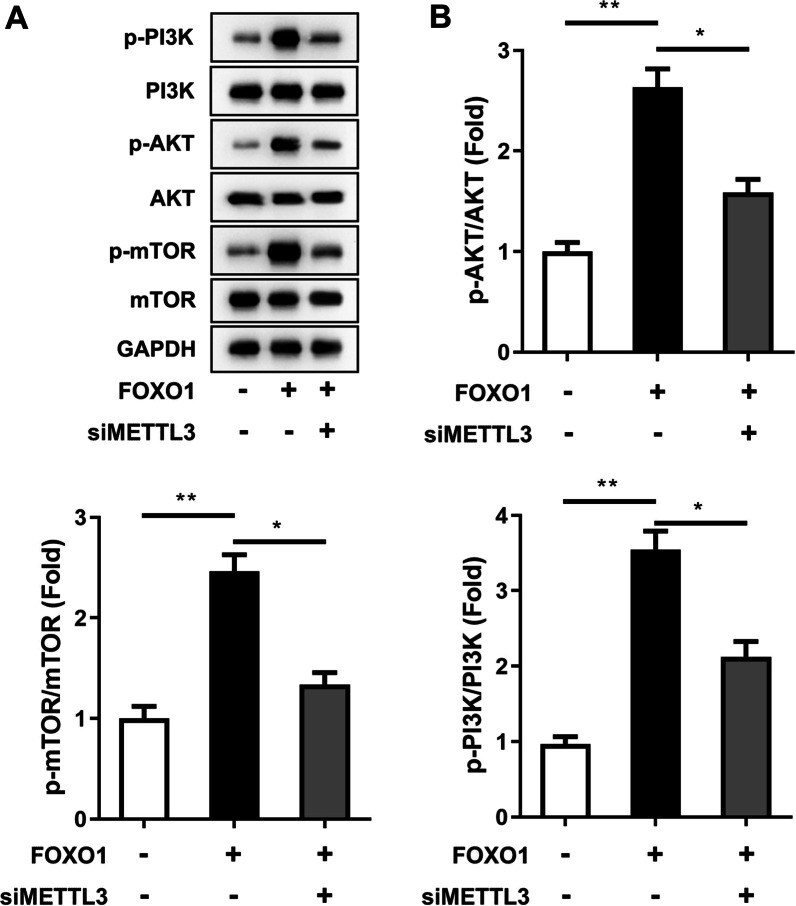
FOXO1 affected the osteogenic differentiation of PDLSCs through the METTL3/AKT/PI3K axis. The cells underwent 14 days of osteogenic differentiation induction. **A** Then, western blotting was used to detect the protein expression levels of AKT, p-AKT, PISK and GAPDH. **B** Quantitative analysis of western blot results. *p < 0.05, **p < 0.01

To further clarify the specific pathway by which FOXO1 promotes the osteogenic differentiation of PDLSCs, we tested the stem cell differentiation-related pathway PI3K/AKT regulated by METTL3. The results showed that the protein expression levels of AKT, p-AKT, and PI3K were increased after overexpression of FOXO1, but knocking down METTL3 reversed this result (Fig. 6A,  B). These results indicated that the PI3K/AKT signaling pathway was activated by the regulation of METTL3 by FOXO1. This finding also indicated that FOXO1 affected the osteogenic differentiation of PDLSCs through the METTL3/AKT/PI3K axis.

## Discussion

Forkhead box O (FOXO) protein is a transcription factor involved in the differentiation of various cell types [30]. FOXO1 is one of the FOXO families. In our study, we investigated the expression and mechanism of FOXO1 during the osteogenic differentiation of PDLSCs. We also verified that FOXO1 can regulate the m^6^A modification of METTL3. This process activated the effect of the PI3K/AKT signaling pathway on the osteogenic differentiation of PDLSCs.

Periodontitis is a chronic inflammation that occurs in periodontal tissue [1, 31]. Worldwide, especially in China, periodontitis has a very high incidence rate [4, 5, 32]. PDLSCs are cells with the ability to differentiate in multiple directions. PDLSCs are also ideal candidates for periodontal tissue and bone regeneration in patients with periodontitis [7–9, 33]. Therefore, identification of drugs and drug targets that promote the osteogenic differentiation of PDLSCs is urgently needed for the clinical treatment of periodontitis patients. Previous studies have shown that FOXO1 regulates the differentiation process of stem cells [34]. On the one hand, FOXO1 dysfunction led to abnormal differentiation of mouse embryonic stem cells [35]. On the other hand, FOXO1 could also regulate the osteogenic differentiation process of human bone marrow-derived mesenchymal stem cells [36]. Wang et al. demonstrated the effect of FOXO1 on the osteogenic differentiation of PDLSCs in patients with periodontitis, which was consistent with our clinical expression and cellular experimental results [16]. In addition, Guo et al. found that FOXO1 can promote osteogenic differentiation of PDLSCs [15]. Our study also confirmed this promotion by detecting calcium nodule formation and osteogenic markers in PDLSCs after overexpression of FOXO1. All these results suggest that FOXO1 is a therapeutic target for periodontitis.

METTL3 is a classic methyltransferase. This molecule participates in m^6^A methylation modification primarily by catalyzing the transfer of methyl to N^6^-adenosine in RNA [37]. This process is one of the core modification methods of m^6^A methylation modification [38]. Therefore, METTL3 can widely participate in the life activities of cells and the occurrence and development of various diseases. Some studies have shown that METTL3 dysfunction can lead to a variety of diseases, such as polycystic kidney disease, osteoarthritis, and chondropathy [39–41]. In recent years, METTL3 has become a leading medical research focus in the treatment of bone-related diseases by promoting the osteogenic differentiation of mesenchymal stem cells and osteoblasts [42, 43]. Cai et al. discovered the regulatory effect of METTL3 on the differentiation of dental pulp stem cells [44]. Zhang et al. found that METTL3 promotes the osteogenic differentiation of PDLSCs in patients with periodontitis through transcriptome microarray detection [22]. Our study also further confirmed the promoting effect of METTL3 by detecting the osteogenic differentiation process of PDLSCs. In addition, we have further elucidated the molecular mechanism by which METTL3 promotes the osteogenic differentiation of PDLSCs through biochemical analysis, FOXO1 expression interference and rescue experiments. Our study showed that the transcription factor FOXO1 transcriptionally activated METTL3 to induce osteogenic differentiation of PDLSCs.

The PI3K/AKT signaling pathway is a classic signaling pathway that regulates the self-renewal and pluripotent differentiation of stem cells. This axis is not only related to phosphatidylinositol but also a signal pathway derived through RTK mediation [45]. Kang et al. demonstrated through transcriptomic analysis and biological evaluation that the PI3K/AKT signaling pathway regulates the osteogenic differentiation of human stem cells [46]. In addition, Li et al. showed that the m^6^A-modifying enzyme ALKBH5 affected the osteogenic differentiation process of mesenchymal stem cells by regulating PI3K/AKT [47]. Our study found that FOXO1 transcription activated METTL3 to regulate the osteogenic differentiation of PDLSCs, which may occur through the activation of the PI3K/AKT pathway.

All the above data indicated that FOXO1 and METTL3 had a positive regulatory effect. Our study showed for the first time that FOXO1 activated the PI3K/AKT signaling pathway by transcriptionally activating METTL3, ultimately promoting the osteogenic differentiation process of PDLSCs.

## Conclusion

In summary, we showed that FOXO1 is generally underexpressed in patients with periodontitis and that FOXO1 expression is associated with the osteogenic differentiation process of PDLSCs. The binding of FOXO1 to METTL3 affected the PI3K/AKT signaling pathway. Therefore, FOXO1 may be a potential target for the treatment of periodontitis. Our research indicated that targeting FOXO1 to promote the osteogenic differentiation of PDLSCs is a potential method for the treatment and prevention of periodontitis. This study has limitations. However, in the future, we will add more clinical data and in vivo experiments to improve the understanding of the mechanism by which FOXO1 regulates the osteogenic differentiation of PDLSCs through the METTL3 signaling pathway.

## Data Availability

The datasets used and/or analyzed during the current study are available from the corresponding author on reasonable request.
